# Abscess of Liver

**Published:** 1874-03

**Authors:** John W. Hayne

**Affiliations:** Tennessee


### ABSCESS OF LIVER.

BY JOHN W. HAYNE, M.D , OF TENNESSEE.

I send you the history of a case of abscess of the liver, that fell under my observation last spring.

April 27th, I was called on to prescribe for a negro man, stout in appearance, aged 27 years, had always enjoyed good health, occupation farming. He complained of a dull pain just beneath the ribs on the right side. The whole surface of the liver appeared somewhat swolen, tongue slightly coated. Believing it to be an ordinary hepatic derangement, I prescribed a brisk mercurial dose, to be followed by two or three pills of quinine on the next day. I was called to visit him on May 1st, learnt that the medicine previously given had brought the expected "dark bilious discharges," but from that date the liver did not seem to act. The region of the liver was smartly swolen. I directed him to take pills composed of calomel, co. ext. colocynth and jalap; these pills to be taken every 2½ hours, until effect was produced. May 3d, I found him suffering intensely with a "dead pain," (as he termed it) in his liver; pulse at about 115, medicine that had been given had little or no effect; tongue very much coated. I then ordered castor oil to be given, and also medicine similar to that previously given, and caused mercurial ointment to be rubed over his entire right side.

May 5th, medicine seemed to have little or no effect on the liver, which by this time was considerably swolen, complained of great and constant pain there, and only there; tongue very foul, pulse 124, no appetite. I placed a large sheet of the fly blistering ointment over his liver, with instructions to let it stay until a good blister was drawn. A few more pills of cholagogue and alterative medicine, to follow the non-effect of the medicine with one or more seidlitz powders.

May 7th, tongue very foul, pulse 130, no appetite. Patient complaining of a gnawing pain incessantly, but little action from the bowels since last visit. Some softness discovered about the center of the liver.

May 9th, accompanied by Dr. Murchison, I found a very perceptable softness, surrounded by a circumscribed wall of resistance, the circumscribed softness 2½ inches in diameter, being about the lower and central portion of the right lobe of the liver. Bowels still obstinate, pulse 130, tongue badly furred. Left alterative medicine, with some dovers powders to alleviate his sufferings.

May 11th, we find the pulse 134, tongue as coated as usual, complaining of intense pain, etc. We placed the patient reclining to his right side, and when under the influence of chloroform I introduced a trochar and canula 2½ inches in the center of the soft circle described, after withdrawing the trochar, I found that the pus was too thick to come off through the canula, I then introduced a medium size abscess lancet all the way down the side of the canula, making a free incision, from which opening, I succeeded in bringing forth a cup full of purulent pus; ordered him to remain on his right side; gave him large doses of opium, wine, brandy, etc.

May 12th, found the patient about the same as regard to feeling pulse 138; aperture in the side closed up, which I reopened about the same way with about the same effect, viz: another cup of purulent matter. From this date, under treatment of opium, stimulants and nourishment, he continued to mend, up the 17th May, when I discharged him. About the middle of June he came a distance of six miles after me, at night, to visit some of his family, and states that he suffers no inconvenience whatever, but has enjoyed the best of health ever since.

---

Fistula in Ano.—Dr. Hute employs an ethereal solution of iodine in fistula in ano, which is more exciting than the tincture. Patients are not obliged to keep their beds. He has had several cures after one injection.—*Medical and Surgical Reporter.*

